# Estimation of Short-term Mortality and Morbidity Attributed to Fine Particulate Matter in the Ambient Air of Eight Iranian Cities

**DOI:** 10.29024/aogh.2308

**Published:** 2018-08-31

**Authors:** Majid Kermani, Gholamreza Goudarzi, Abbas Shahsavani, Mohsen Dowlati, Farshad Bahrami Asl, Sima Karimzadeh, Sevda Fallah Jokandan, Mina Aghaei, Babak Kakavandi, Babak Rastegarimehr, Sasan Ghorbani-Kalkhajeh, Ramin Tabibi

**Affiliations:** 1 Research Center for Environmental Health Technology, Iran University of Medical Sciences, Tehran, IR; 2 Department of Environmental Health Engineering, School of Public Health, Iran University of Medical Sciences, Tehran, IR; 3 Department of Environmental Health Engineering, Ahvaz Jundishapur University of Medical Sciences, Ahvaz, IR; 4 Department of Environmental Health Engineering, School of Public Health, Shahid Beheshti University of Medical Sciences, Tehran, IR; 5 Department of Environmental Health Engineering, School of Public Health, Hamadan University of Medical Sciences, Hamadan, IR; 6 Department of Environmental Health Engineering, School of Public Health, Urmia University of Medical Sciences, Urmia, IR; 7 Center for Air Pollution Research (CAPR), Institute for Environmental Research (IER), Tehran University of Medical Sciences, Tehran, IR; 8 Research Center for Health, Safety and Environment, Alborz University of Medical Sciences, Karaj, IR; 9 Department of Environmental Health Engineering, Alborz University of Medical Sciences, Karaj, IR; 10 Abadan School of Medical Sciences, Abadan, IR

## Abstract

Amongst the various pollutants in the air, particulate matters (PM) have significant adverse effects on human health. The current research is based on existing epidemiological literature for quantitative estimation of the current health impacts related to particulate matters in some selected principal Iranian megacities. In order to find the influence of air pollution on human health, we used the AirQ software tool presented by the World Health Organization (WHO) European Centre for Environment and Health (ECEH), Bilthoven Division. The adverse health outcomes used in the study consist of mortality (all causes excluding accidental causes), due to cardiovascular (CVD) and respiratory (RES) diseases, and morbidity (hospital admissions for CVD and RES causes). For this purpose, hourly PM_10_ data were taken from the monitoring stations in eight study cities during 2011 and 2012. Results showed annual average concentrations of PM_10_ and PM_2.5_ in all megacities exceeded national and international air quality standards and even reached levels nearly ten times higher than WHO guidelines in some cities. Considering the short-term effects, PM_2.5_ had the maximum effects on the health of the 19,048,000 residents of the eight Iranian cities, causing total mortality of 5,670 out of 87,907 during a one-year time-period. Hence, reducing concentrations and controlling air pollution, particularly the presence of particles, is urgent in these metropolises.

## Introduction

Air pollution is considered a major environmental risk to human health, causing both acute and chronic respiratory illnesses [1]. The evidence for particulate matters (PM) and its public health impacts indicate adverse health effects at exposure to concentrations currently found in many cities in developed and developing countries [2,3,4]. The range of effects on human health is wide, but they mostly include respiratory and cardiovascular diseases [5,6,7]. Among the air pollutants, particulate matters have the most negative effects on human health. This pollutant is of paramount importance due to the way it penetrates into the lower respiratory tract [8,9].

In the past few decades, many investigations have indicated a direct relationship between the presence of particulate matters and various diseases, such as cardiopulmonary mortality [10,11,12], respiratory hospitalizations [13,14], lung function and respiratory symptoms, mortality and hospitalization [15]. Some researchers believe there is not strong evidence of health effects at low-to-moderate particulate pollution levels [16,17,18], but others argue there are negative influences on human health at either low or high concentrations [19,20].

Air pollution and concerns about it are increasing, particularly in developing countries. Individuals in megacities encounter air pollution every day [2,21,22]. In the present study, eight Iranian cities with a total population of 19 million inhabitants in different provinces were investigated: Tehran, Mashhad, Tabriz, Isfahan, Shiraz, Ahwaz, Arak and Urmia. These eight cities hold 26.6% of the Iranian population. These megacities are affected by increasing air pollution levels as a result of industrial activities, urbanization, heavy traffic and high population density. Thus, the main aim of this research is to evaluate the short-term human health impact of fine particles with an aerodynamic diameter of less than 10 μm (PM_10_) and 2.5 μm (PM_2.5_) in eight metropolitan cities of Iran during 2011 and 2012.

## Method

**Figure 1:**
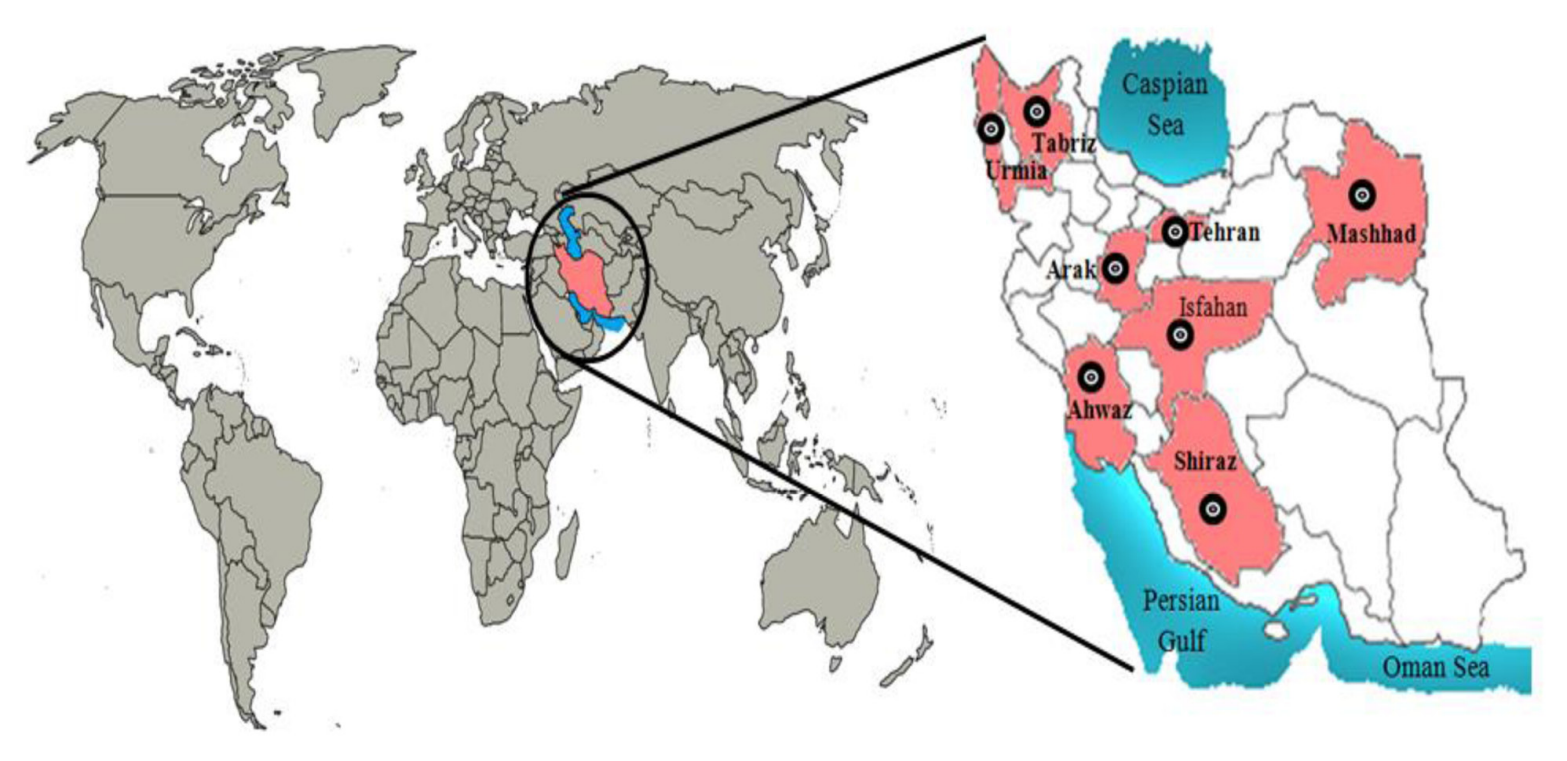
Map of Iran in the world and location of studied metropolises (studied cities given by black dots).

The current epidemiological study attempts to estimate the health impacts attributable to particulate matters and is a type of cohort study in epidemiological classification implemented by the AirQ model. Data were collected from air pollution monitoring stations operated by the Iranian Environmental Protection Agency (EPA) in eight major cities of Iran (i.e., Tehran, Mashhad, Tabriz, Isfahan, Shiraz, Ahwaz, Arak and Urmia). The location of these cities is shown in Figure 1. The population of studied cities was adopted from the recent census report issued by the Statistical Centre of Iran (SCI) in 2011 (Table 1). According to the criteria for the Air Quality Health Impact Assessment, the monitoring stations with valid data were identified and investigated. Air pollution data included a 24-hour average measurement of particles with a diameter less than 10 μm (PM_10_) and 2.5 μm (PM_2.5_).

**Table 1:** Population (SCI 2011), Latitude and longitude of eight major Iranian cities.

City	Exposed population*	Latitude	Longitude

Tehran	9000000	35.34	51.25
Mashhad	2750000	36.31	59.58
Tabriz	1495000	38.08	46.28
Isfahan	1987000	32.68	51.64
Shiraz	1540000	29.62	52.52
Ahwaz	1112000	31.32	48.68
Arak	484000	34.09	49.7
Urmia	680000	37.55	45.07
Total population	19048000	**–**	**–**

* According to the report of statistical center of Iran.

### Health impact assessment tool

To quantify the impact of air pollution on human health in eight cities of Iran, we applied the Air Quality Health Impact Assessment (AirQ) tool (AirQ2.2.3 software) developed by the WHO European Centre for Environment and Health, Bilthoven Division in 2004. A broad variety of specific outcomes or health endpoints are considered for assessing human health impact. The AirQ also provides useful information about the potential impacts of exposure to a given air pollutant on human health for a defined urban area within a defined period of time [23]. We applied the Iranian EPA data to provide an input file for an air quality screen of the AirQ model. The stations’ data were preprocessed in Excel to convert the data into the AirQ input format. The collected data included daily average, annual mean, winter and summer days, annual, winter (October to March) and summer (April to September) mean, annual, winter and summer maximum, Annual 98 percentile, the number of days concentrations of the air pollutants were in certain intervals and population divided by one thousand.

### Data analysis

**Figure 2:**
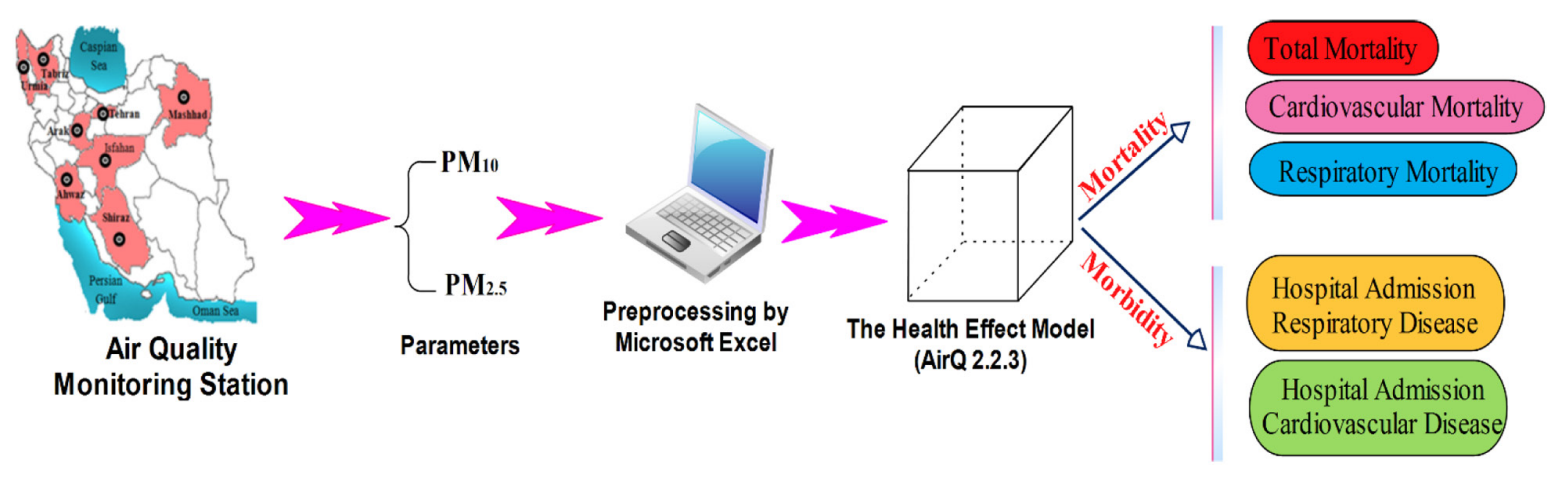
Schematic applied in this study for data analysis.

AirQ2.2.3 software was used to assess the health impact of PM_10_ and PM_2.5_ exposure in the studied cities. The schematic applied is illustrated in Figure 2. This software has been employed in a variety of past studies [23,24] and allows for the calculation of possible effects of exposure to air pollutants in specific urban areas during a certain time period. The adverse health outcomes in the study consist of mortality (all causes excluding accidental causes), due to cardiovascular (CVD) and respiratory (RES) disease, and morbidity (hospital admissions due to CVD and RES causes). This program was based on a risk assessment approach and estimates the human health effect of exposure to special atmospheric contaminants in a specific region. Attributable proportion (AP) was the most important parameter in the software, which was calculated based on relative risks (RR). AP is the ratio of health outcomes in a specific population (in a specific area and period) attributed to air pollutant exposure, meaning there is a causative association between exposure to the air pollutant and health consequences and there are no significant factor effects regarding such association [23,25]. AP was obtained according to Equation 1. RR is defined as the relative risk for health consequences in category *c* of exposure, taken from the exposure–response functions obtained from epidemiological research results, and *p(c)* denotes the proportion of the target population in category *c* of exposure [23,25]. RR is the health attributable risk to exposed people and is computed by Equation 2.

1 AP = \sum {\{[RR(c) - 1] \times p(c)\}/\sum {[RR(c) \times p(c)]}}

2 RR = \left[{\frac{\begin{array}{c}
{\rm{Probability\,of\,event\,when\,exposed}}\\
{\rm{to\,air\,pollution}}
\end{array}}{\begin{array}{c}
{\rm{Probability\,of\,event\,when\,not\,exposed}}\\
{\rm{to\,air\,pollution}}
\end{array}}}\right]

If the baseline incidence of the health endpoint in the studied population was known, the health outcome rate related to the exposure was obtained via the following equation:

3 IE = I \times AP

where IE is defined as a number of reported health outcomes related to the exposure and I is defined as a baseline incidence of the health impact on the studied population [13,26]. Regarding the population, the quantity of cases related to exposure was obtained according to the following formula:

4 NE = IE \times N

where NE is the quantity of cases related to exposure to a specific pollutant and N is the size of the investigated population.

RR values applied in this assessment are reported in Table 2. For PM_10_ and PM_2.5_, RR values were obtained from a quantitative meta-analysis in the literature [27]. To prevent underestimating and overestimating the short-term impact of air pollutants, the upper and lower estimates were calculated by applying the upper and lower coefficients of the confidence intervals to estimates of the relative risks. All coefficients were represented based on a relative risk per 10μg/m^3^ increase in PM_10_ and PM_2.5_ concentrations. Baseline incidence (BI) rates for the health impact were expressed per 105 populations per year [28]. The rates for all deaths were obtained from similar studies conducted in Iran and in terms of another health endpoint, such as hospital admissions, proposed by the WHO.

**Table 2:** Relative risk with 95% confidence intervals and Baseline Incidence per 10^5^ persons for each health impact estimates in the present study.

Health endpoint		Baseline incidence^a^	PM_10_ RR (95% CI) per 10 μg/m^3^	PM_2.5_ RR (95% CI) per 10 μg/m^3^

Mortality	Death (all cases) ICM^b^-9-CM o800	543.5	1.006 (1.004–1.008)^c^ (Anderson *et al*., 2004; Fattore *et al*., 2011)	1.015 (1.011–1.019) (Fattore *et al*., 2011; Organization. 2001)
	Cardiovascular disease ICM-9-CM 390–459	231	1.009 (1.005–1.013) (Anderson *et al*., 2004; Fattore *et al*., 2011)	–
	Respiratory disease ICM-9-CM 460–519	48.4	1.013 (1.005–1.020) (Anderson *et al*., 2004; Fattore *et al*., 2011)	–

Morbidity	HA^d^ for cardiovascular disease	436	1.009 (1.006–1.013) (Martuzzi *et al*., 2002; Organization. 2001)	–
	HA for respiratory disease	1260	1.008 (1.0048–1.0112) (Touloumi *et al*., 1996)[30]	–

^a^ Crude rate per 100,000 inhabitants. ^b^ International Classification of Diseases. ^c^ Daily Average. ^d^ Hospital Admission.

## Results

**Figure 3:**
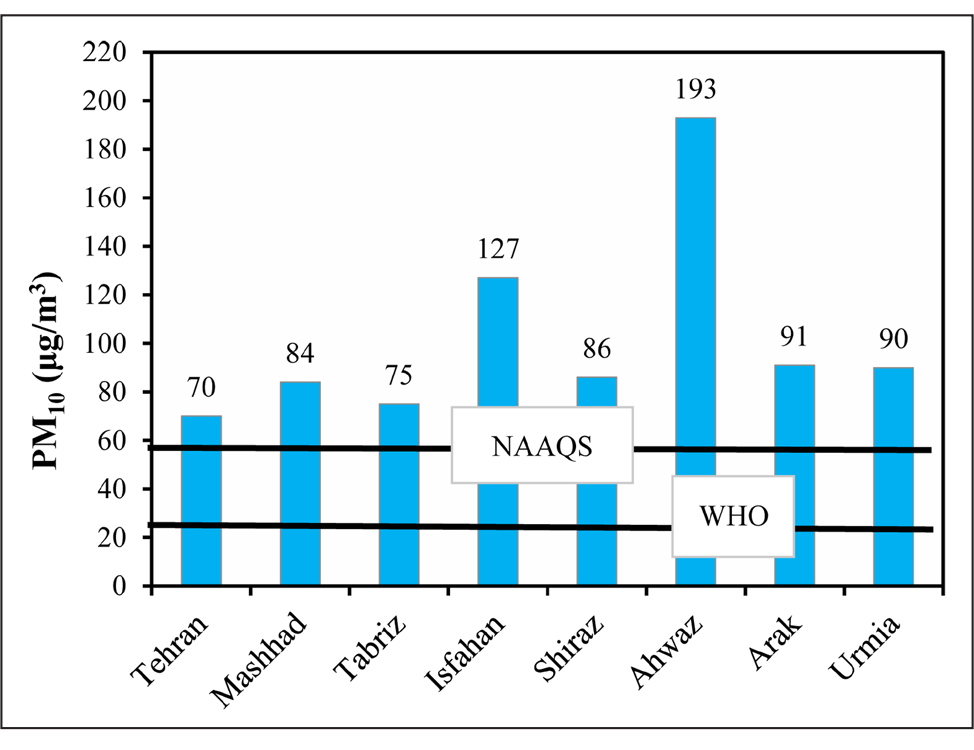
Annual mean variations of PM_10_ concentration (μg/m^3^) in 8 megacities based on average data.

**Figure 4:**
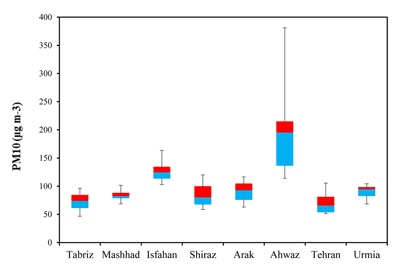
Summary of descriptive statistics of PM_10_ concentrations (μg/m^3^) measured in 8 megacities stations during 2011 to 2012 (as a monthly average).

For particulate matters, the required statistical parameters (annual and seasonal maximum and annual 98th percentiles) were obtained in each city. The summary of the statistics of PM_10_ and PM_2.5_ are represented in Tables 3 and 4, respectively. Initially, the concentration of PM_10_ and PM_2.5_ in eight Iranian cities were analyzed and compared with air quality guidelines and standards (Table 5). In the present study, the annual mean concentrations of PM_10_ and PM_2.5_ in Isfahan were 127 and 76μg/m^3^, respectively, which were approximately 5 to 6.5 times higher than the WHO air quality guidelines. Figure 3 shows the annual profile of PM_10_ level (μg/m^3^). Maximum concentrations of PM_10_ were recorded in the winter season in Ahwaz, Isfahan, Mashhad and Urmia. Among the investigated cities, the highest annual concentration of PM_10_ (μg/m^3^) was observed in Ahwaz, with an annual maximum and mean value of 2521 and 193μg/m^3^, respectively. Results showed that 24-hour averages of PM_10_ were 304, 254, 321, 306 and 228 days higher than the WHOs standards in Mashhad, Tabriz, Isfahan, Shiraz and Urmia, respectively. A summary of descriptive statistics of PM_10_ concentrations measured in eight megacities is shown in Figure 4. In other cities, such as Tabriz and Mashhad, the annual mean concentrations of PM_10_ were 3.75 and 4.2 times higher than WHO standards. Results revealed that PM_10_ and PM_2.5_ levels in all megacities exceeded national and international air quality standards and guidelines set and proposed for the protection of human health.

**Table 3:** PM_10_ concentrations (μg/m^3^) in eight megacities during 2011 to 2012.

Parameter	Tehran	Mashhad	Tabriz	Isfahan	Shiraz	Ahwaz	Arak	Urmia

Annual mean	70	84	75	127	86	193	91	90
Winter mean^1^	62	85	70	115	87	185	81	83
Summer mean^2^	79	82	80	138	93	198	102	96
Annual 98 Percentile (P_98_)	144	180	218	225	217	742	208	233
Annual maximum	289	296	400	337	330	2521	471	683
Winter maximum	169	296	400	254	330	2521	323	156
Summer maximum	289	277	321	337	294	764	471	683
No. of station^3^	12	4	4	4	2	1	1	1
Data capture (Day)	365	362	365	365	362	262	351	270

^1^ Winter cool season: October to March. ^2^ Warm season: April to September. ^3^ Number of monitoring stations with valid data.

**Table 4:** PM_2.5_ (μg/m^3^) annual maximum concentration in the eight Iranian cities during 2011 to 2012.


Tehran	42	Shiraz	51
Mashhad	50	Ahwaz	115
Tabriz	45	Arak	55
Isfahan	76	Urmia	54


**Table 5:** Standards and guidelines for average ambient particulate concentration (μg/m^3^).

Standard or guideline	PM_10_ (μg/m^3^)		PM_2.5_ (μg/m^3^)	

	annual	24 hours	annual	24 hours

WHO guidelines (WHO 2005)	20	50	10	25
National Ambient Air Quality Standards(NAAQS)	50	150	25	35
Iran national standard	20	50	10	25
State of California	20	–	12	–
Other European countries	20		As low as possible	
U.S.A Federal standard	–	–	12	–

**Figure 5:**
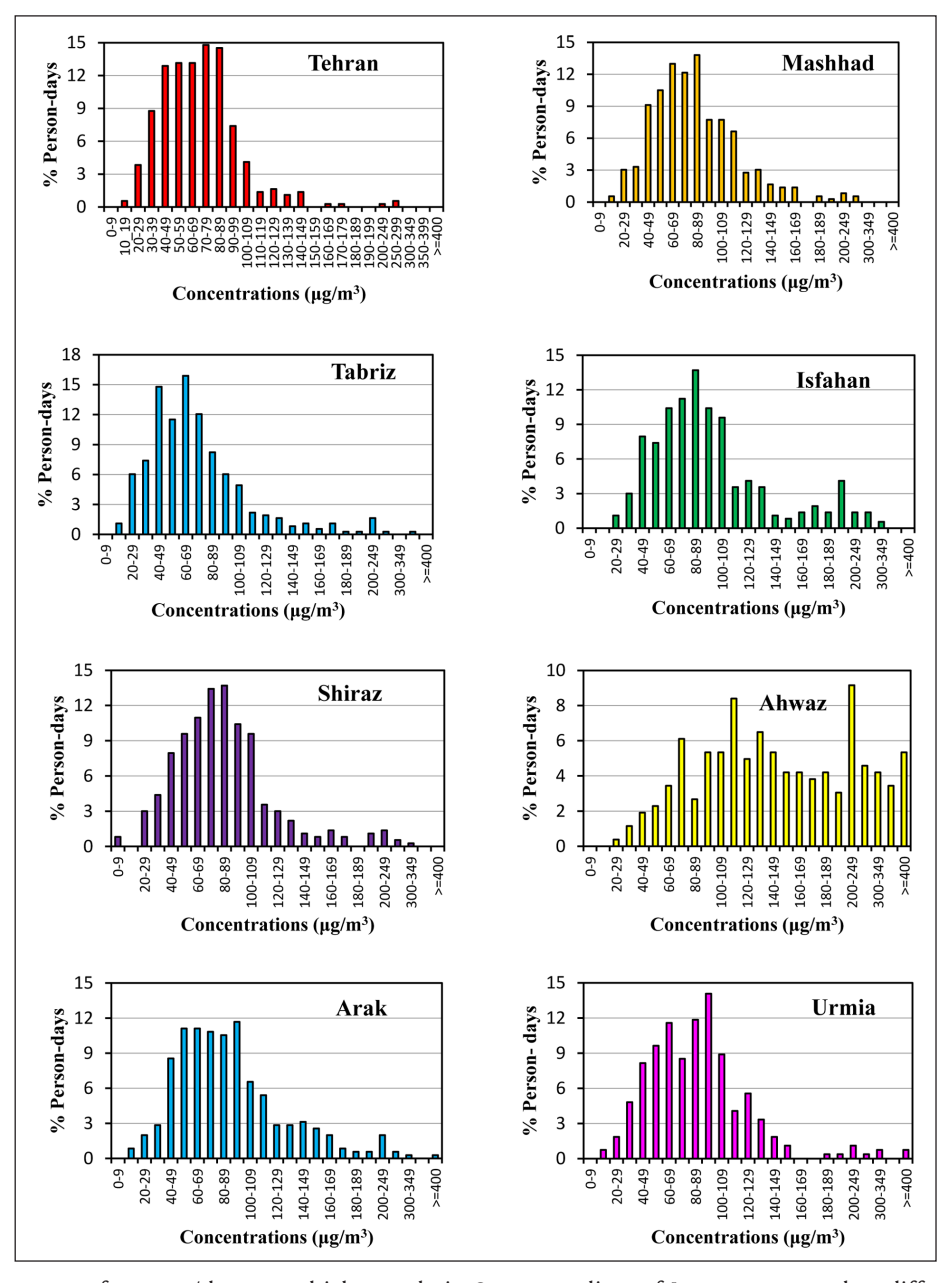
Percentage of person/days on which people in 8 metropolises of Iran are exposed to different concentrations of PM_10_.

Figure 5 shows the concentration interval of PM_10_ and the time percentage of individual exposure to these levels. In all cities, except Ahwaz, the highest percentage of person/days was associated with concentrations less than 100μg/m^3^ of PM_10_. The maximum person/day percentage, according to the AirQ table, was in Tabriz (15.89% in concentration ranges of 60–69μg/m^3^), Tehran (14.79% in 70–79μg/m^3^), Mashhad (13.81% in 80–89μg/m^3^), Isfahan and Shiraz (13.70% in 80–89μg/m^3^), Arak (11.68% in 90–99μg/m^3^) and Urmia (14.07% in 90–99μg/m^3^). In Ahwaz, it was higher than 100μg/m^3^ (9.16 % in 200–249μg/m^3^). The obtained results showed the effects of PM_10_ and PM_2.5_ in ambient air on the residents’ health as measured by attributable cases for the selected outcomes. The short-term influence of PM_10_ exposure on human health during 2011 and 2012, which was higher than the reference value of 10μg/m^3^, is summarized in Table 6. Accordingly, the number of excess hospitalizations for cardiovascular and respiratory diseases was attributed to PM_10_. Health impacts were determined to be increasing in all cases: cardiovascular and respiratory mortality and hospital admission for cardiovascular and respiratory diseases due to short-term exposure to PM_10_ above a reference value of 10μg/m^3^. Based on the obtained findings, the estimated total cumulative number of deaths due to all cases for eight cities was 4402 out of 90,205 people in a year. In terms of total mortality, the highest impact attributed to PM_10_, with an AP of 9.07%, corresponded to an excess of 549 cases in Ahwaz. There were 13,402 hospital admissions for respiratory diseases (6.40%) related to PM_10_, and 7.11%, or 5177, extra cases of CVD. Respiratory mortality was the endpoint with the highest attributable proportion, reaching 17.78% in Ahwaz. Overall, 811 attributable cases were estimated, which equals 9.63%. PM_10_ is responsible for 7.14% of all mortality as a result of cardiovascular causes in all the studied cities, which equals 2742 deaths. In all calculated endpoints, Ahwaz city has the greatest AP.

**Table 6:** Estimated attributable proportion (AP) expressed as percentage and number of excess cases in a year due to short-term exposure per 10μg/m^3^ increase in the concentration of PM_10_.

Health Endpoints	City	AP (uncertainty range)	No. of excess cases (uncertainty range)

Total mortality (TM)	Tehran Mashhad Tabriz Isfahan Shiraz Ahwaz Arak Urmia	3.51 (2.37–4.63) 4.24 (2.87–5.58) 3.71 (2.5–4.89) 5.02 (3.4–6.58) 4.33 (2.92–5.69) 9.07 (6.24–11.74) 4.67 (3.17–6.14) 4.56 (3.08–5.99)	1721 (1161–2268) 634 (429–834) 302 (204–398) 542 (368–711) 362 (245–476) 549 (377–710) 123 (83–161) 169 (114–221)

Cardiovascular mortality (CM)	Tehran Mashhad Tabriz Isfahan Shiraz Ahwaz Arak Urmia	5.18 (2.94–7.32) 6.23 (3.56–8.76) 5.47 (3.11–7.71) 7.34 (4.22–10.28) 6.36 (3.63–8.93) 13.02 (7.68–17.78) 6.85 (3.92–9.6) 6.69 (3.83–9.38)	1078 (613–1522) 396 (226–557) 189 (108–266) 337 (194–472) 226 (129–318) 335 (197–457) 77 (44–107) 105 (60–148)

Respiratory mortality (RM)	Tehran Mashhad Tabriz Isfahan Shiraz Ahwaz Arak Urmia	7.32 (2.94–10.83) 8.76 (3.56–12.87) 7.71 (3.11–11.4) 10.28 (4.22–14.98) 8.93 (3.63–13.11) 17.78 (7.68–24.96) 6.9 (3.92–14.05) 9.38 (3.83–13.74)	319 (129–472) 118 (48–173) 56 (23–83) 100 (41–145) 67 (27–98) 97 (42–135) 23 (9–33) 31 (13–46)

Hospital Admissions Cardiovascular Disease (HACD)	Tehran Mashhad Tabriz Isfahan Shiraz Ahwaz Arak Urmia	5.18 (3.51–7.32) 6.23 (4.24–8.76) 5.47 (3.71–7.71) 7.37 (5.02–10.27) 6.36 (4.33–8.93) 13.02 (9.07–17.78) 6.58 (4.67–9.6) 6.69 (4.56–9.38)	2035 (1381–2873) 747 (509–1050) 357 (242–502) 637 (435–890) 427 (291–600) 631 (440–862) 145 (99–202) 198 (135–278)

Hospital Admissions Respiratory Disease (HARD)	Tehran Mashhad Tabriz Isfahan Shiraz Ahwaz Arak Urmia	4.63 (2.83–6.37) 5.58 (3.42–7.64) 4.89 (2.99–6.72) 6.58 (4.05–8.98) 5.69 (3.49–7.79) 11.74 (7.39–15.7) 6.14 (3.77–8.39) 5.99 (3.68–8.19)	5258 (3215–7228) 1933 (1186–2648) 922 (562–1266) 1649 (1016–2249) 1105 (678–1512) 1646 (1036–2201) 375 (230–512) 514 (316–702)

The adverse health outcomes used for PM_2.5_ in the current study was mortality (all causes, except external causes/excluding accident). The short-term effects of PM_2.5_ exposure above a reference value of 10μg/m^3^ during 2011 to 2012 is summarized in Table 7. Considering short-term exposure, the maximum influence for total mortality among these cities belonged to PM_2.5_, with an AP of 13.01%, which led to 787 cases in Ahwaz city. In addition, similar results were obtained for total mortality of PM_10_ in Ahwaz city, compared to the other studied cities, as represented in Table 6. Therefore, the attributable proportion of 9.07% was obtained from concentrations in excess of 10μg/m^3^ that caused about 549 cases of total mortality. In Tehran, 4.56% of all deaths are attributable to PM_2.5_ concentrations in excess of 10μg/m^3^, causing about 2232 cases of total mortality, while 3.51% of deaths, or 1721 cases, were attributable to PM_10_. For this health endpoint, the attributable proportion in Arak city was 6.26%, or 165 cases. In total, PM_2.5_ seems to be responsible for 6.45% of all deaths (excluding accidental causes) in all the studied cities. Around 5670 attributable numbers were obtained during 2011 to 2012. In contrast, for PM_10_ the effect was estimated at 4402 extra cases yearly with an AP of 4.48%.

**Table 7:** Estimated attributable proportion (%AP) and mortality attributable to short-term exposure to PM_2.5_ concentration above 10μg/m^3^ (excluding accident causes) in 8 Iranian cities.

	Estimated no. of cases	95% CL		Estimated % of cases	95% CL			

Tehran	2232	1657	2793	4.56	3.38	^a^	5.71	^b^
Mashhad	754	560	942	5.04	3.74		6.3	
Tabriz	463	345	578	5.7	4.26		7.11	
Isfahan	585	435	731	5.42	4.03		6.76	
Shiraz	454	338	567	5.42	4.04		6.77	
Ahwaz	787	598	963	13.01	9.88		15.93	
Arak	165	123	205	6.26	4.67		7.8	
Urmia	230	171	286	6.21	4.63		7.74	
Total	5670			6.45				

^a^ Obtained using the lower RR values. ^b^ Obtained using the upper RR values.

## Discussion

Ahwaz, the capital of Khuzestan province and the most polluted city in the world, is located in southwestern Iran, which has witnessed heavy dust storms during the last decade [2]. In comparison with the standards, the annual mean of PM_10_ in Ahwaz city was 9.65 times higher than the WHO air quality guideline values, while this value in Arak, Shiraz, Isfahan and Urmia was approximately 4.5 to 6 times higher than the mentioned guideline (20μg/m^3^), as given in Table 3. On the other hand, the differences in the annual mean of PM_10_ concentrations of Ahwaz city with Tehran, Mashhad, Tabriz, Isfahan, Shiraz, Arak and Urmia were 123, 109, 118, 66, 107, 102 and 103μg/m^3^, respectively, which are apparently remarkable. Furthermore, a significant difference can be observed when the PM_2.5_ (μg/m^–3^) annual maximum concentration of Ahwaz city is compared with the other cities, according to the results in Table 4. The high concentrations of PM in Ahwaz could be associated with its arid nature and dust events, as well as its geographic position close to the deserts of Iraq, Saudi Arabia and Kuwait, which are known major sources of particulate matters [31,32].

According to the results in Tables 6 and 7, it can be concluded that the mortality attributable to PM_2.5_ is significantly greater than PM_10_, indicating that PM_2.5_ has the higher health effects compared to PM_10_ in all the studied cities, which is in good agreement with findings reported in the literature [10,23].

Our study estimated thousands of deaths, hospital admissions, and cases of respiratory and cardiovascular diseases in the eight largest Iranian cities associated with particulate matters. Similarly, the conducted research within the last years confirmed that ambient air pollution contributed to morbidity and mortality [12,15,23,29,33,34,35,36,37] (Table 8).

**Table 8:** Summary of similar studies conducted in this field.

Study (city)	Author, Year	Results

		Attributable number of cases to PM Health outcomes

Two areas of Northern Italy	(Fattore *et al*., 2011)	In this study, PM_2.5_ had the highest health impact on the 24,000 inhabitants that caused an excess of total mortality of 8 out of 177 in a year.
Makkah	(Habeebullah, 2013)	The cumulative number of estimated average hospital admissions due to respiratory illnesses during the study period was 112,665 per 10μg/m^3^ increase of PM_10_ concentration.
U.S. 6 cities	(Laden *et al*., 2000)	In the combined analysis across the six cities, controlling for other sources, a 10μg/m^3^ increase in PM_2.5_ from mobile sources accounted for a 3.4% increase in daily mortality (CI, 1.7–5.2%).
Eight European cities	(Le Tertre *et al*., 2002)	Percentage increases associated with a 10μg/m^3^ increase in PM_10_ and 0.5% (95% CI: 0.2 to 0.8) for cardiac admissions of all ages.
Eight major Italian cities	(Martuzzi *et al*., 2002)	Results indicated that 4.7% of mortality (95% CI, 1.7–7.5) is attributable to PM_10_ concentrations higher than 30μg/m^3^. The numbers of attributable deaths were 3472.
23 Italian cities	(Boldo *et al*., 2006)	The HIA estimated that 16,926 premature deaths from all causes, including 11,612 cardiopulmonary deaths and 1901 lung-cancer deaths, due to PM_2.5_ long-term exposure.
Ulaanbaatar, Mongolia	(Allen *et al*., 2013)	Estimated that 29% (95% CI, 12–43%) of cardiopulmonary deaths and 40% (95% CI, 17–56%) of lung cancer deaths in the city are attributable to outdoor air pollution.
13 Italian cities	(Martuzzi *et al*. 2006)	Considering the short-term effects on mortality (within a week after exposure), the impact of PM_10_ above 20μg/m^3^ was 1372 deaths or 1.5% of the total mortality in the whole population.
European assessment (Austria, France and Switzerland)	(Künzli *et al*., 2000)	A study conducted in Austria, France and Switzerland has estimated air pollution caused 6% of total mortality, or more than 40,000 attributable cases, per year to PM_10_ in the 3 countries.

Regarding the short-term impacts, PM_2.5_ could cause the largest health effects for the 19,048,000 residents of the eight Iranian cities, leading to an excess of total mortality of 5670 out of 87,907 during a year. Findings indicate that the adverse health effects of air pollutants currently experienced by urban populations in studied areas require urgent measures by government and urban air quality control authorities paying more attention to air pollution control. Results demonstrated that the highest annual average concentration of PM_10_ was in Ahwaz and Isfahan, with 193 and 127μg/m^3^, respectively. Because of high attributable proportion (AP), the highest total number of cases for total mortality (TM), cardiovascular mortality (CM), respiratory mortality (RM), hospital admissions derived from cardiovascular diseases (HACVD), and hospital admissions due to respiratory diseases (HARD) in central relative risk were in Ahwaz. The health outcomes of particulate matters in urban areas are high due to their large concentration. Developed and extended Iranian cities have annual averages of PM_10_ and PM_2.5_ that exceed national and international air quality standards and guidelines and can reach levels nearly 10 times higher than WHO guidelines in some areas. Therefore, reducing concentrations and controlling air pollution are of paramount of importance.

There are several limitations in the estimation of health impacts related to air pollution. First, estimated health impacts can result both from particulate matters and from other correlated pollutants (e.g., synergistic effects). We estimated without considering these synergistic effects. Hence, the general impact of air pollution is considerably overlooked. Indeed, the applied approaches for this research provide an assessment that describes at least part of the true health effect, but it is likely to be greater [26,29]. Second, some individuals or segments of the population are more susceptible to particulate matter exposure due to various factors, such as respiratory habits, preexisting diseases or genetics [3]. Despite all these complications, fixed monitoring stations at urban sites are used to count total exposure of an individual to PM for whole cities and people residing in the region. Third, relative risks used for estimating health impacts of PM_10_ and PM_2.5_ were based on program defaults and on studies performed in other countries. Health effect assessment studies have to be improved due to air pollutants. Future research could improve the methodology and the quality and integrity of findings, as well as the characterization of all appropriate health outcomes, pay more attention to all contaminants, consider all vulnerable subgroups, and more reliably determine uncertainties [29]. Some argue that estimates of the influence of air pollution on human health rely significantly on the quality and presence of biomedical science and information, and although there are gaps in scientific knowledge in terms of the role of air pollution on human health, the available information does not require preventive action for protecting public health [39]. Finally, the estimation of health endpoints in this survey are related to the particulate matter impacts in the eight largest cities of Iran; for estimating the total national burden of pollution, extra monitoring and modeling may be required all over the country.

## Conclusion

This research evaluated the role of particulate matters on human health in eight megacities of Iran. A study was done to determine the health impact of particles with aerodynamic diameters equal or less than 10 μm (PM_10_) and 2.5 μm (PM_2.5_) in eight metropolitans of Iran in 2011 and 2012. Our findings were similar to previous research on the role of air quality on the human body. AirQ software would be a simple and efficient device, and also a suitable approach, for policy-makers. Quantifying the health impacts associated with exposure to particulate matters and other air pollutants would potentially be an essential tool for a legislator. These estimates will confirm the disastrous aspect of air pollution, so that the reduction and control of air pollution would be concentrated on to enhance public health. This quantification can be applied as an index for determining the required efforts for control.
